# HSP90AA1 promotes the inflammation in human gingival fibroblasts induced by *Porphyromonas gingivalis* lipopolysaccharide via regulating of autophagy

**DOI:** 10.1186/s12903-022-02304-0

**Published:** 2022-08-26

**Authors:** Huang Zhang, Jie Huang, XuSheng Fan, RuiJing Miao, YongWu Wang

**Affiliations:** Department of Stomatology, Affiliated Hangzhou First People’s Hospital, Zhejiang University School of Medicine, Hangzhou, 310006 People’s Republic of China Zhejiang Province

**Keywords:** HSP90AA1, Human gingival fibroblasts, Autophagy, Peri-implantitis, *Porphyromonas gingivalis* lipopolysaccharide

## Abstract

**Background:**

Peri-implantitis of tooth seriously affects the life quality of patients. This study aimed to investigate the role of HSP90AA1 in the inflammatory of human gingival fibroblasts (HGFs) induced by *porphyromonas gingivalis* lipopolysaccharide (Pg-LPS), and to provide a potential therapeutic target for clinical treatment of peri-implantitis.

**Methods:**

Pg-LPS (0.1, 1, 10 μg/mL) was used to construct the inflammatory model of HGFs to evaluate the effect of Pg-LPS on HGFs. Then HSP90AA1-siRNA was transfected to construct HSP90AA1 low expression HGFs cell line, and 3-MA was also added. After that, cell viability, apoptosis, the contents of inflammatory cytokines were detected by CCK-8, flow cytometry and ELISA assay, respectively. Intracellular ROS, the expressions of HSP90α, HSP90β were detected by immunofluorescence. The levels of HSP90AA1, p-NF-κB p65/NF-κB p65, LC3 II/I, ATG5, Beclin-1 and TLR protein were detected by western blot.

**Results:**

Pg-LPS treatment didn’t affect the viability of HGFs cells, but induced the cell apoptosis and ROS generation, increased the contents of IL-1β, IL-6, TNF-α, and the protein expressions of HSP90AA1, p-NF-κBp65/NF-κBp65, LC3II/I, ATG5, and Beclin-1 in HGFs. While HSP90AA1-siRNA transfected into Pg-LPS induced HGFs significantly reduced the HSP90AA1, HSP90α, HSP90β expression, decreased the inflammatory factors, ROS generation, cell apoptosis rate, and autophagy-related proteins and TLR2/4 protein levels. What’s more, the addition of autophagy inhibitor 3-MA further promote the effect of HSP90AA1-siRNA on Pg-LPS treated HGFs.

**Conclusions:**

This study showed that HSP90AA1 promoted the inflammatory response of Pg-LPS induced HGFs by regulating autophagy. The addition of 3-MA further confirmed that autophagy may mediate siHSP90AA1 to enhance the inflammatory response.

**Supplementary Information:**

The online version contains supplementary material available at 10.1186/s12903-022-02304-0.

## Background

Because of its advantages of not damaging adjacent teeth and good retention support, dental implants have changed the prosthetics and treatment methods of some completely or partially edentulous patients, and gradually replaced the position of fixed bridges [1, 2]. Although dental implants had a high success rate, complications and failures were inevitable. Peri-implantitis has been described as a destructive inflammatory lesion that affected the osseointegration of the hard and soft tissues of the implant, resulting in bone loss and peri-implant pocketing [3]. The occurrence of peri-implantitis was related to microbial infection, and the invasion of pathogenic bacteria could lead to a persistent inflammatory response in the gingiva and its surrounding combined alveolar bone. In the late stage of inflammation, there would be absorption of peri-implant pocketing and surrounding alveolar bone, which would eventually cause implant loosening and falling off, resulting in implant failure [4]. In addition, risk factors such as smoking, diabetes, poor oral hygiene, history of periodontitis, improper repair design would also promote the occurrence of peri-implantitis [5, 6]. According to the survey, the incidence rate of peri-implantitis was about 10% [7]. Therefore, studying the pathogenesis of peri-implantitis is of great significance for relieving dental implant complications and improving the quality of life of implant patients.

Human gingival fibroblasts (HGFs) are the main cells of periodontal soft tissue [8]. HGFs played an active and key role in mechanisms of the host immune defense against multiple pathogens, and maintained tissue structure and function [9]. It was reported that bacterial colonization was found when the implant surface was exposed to the mouth for more than 30 min [10]. The main pathogens of peri-implantitis were mainly Gram-negative bacteria, such as *Porphyromonas gingivalis* [11], and the average colony forming units of bacteria in the peri-implantitis sites were higher than that in the healthy sites. *Porphyromonas gingivalis* had multiple virulence factors, such as lipopolysaccharide (LPS), which could cause the metabolism and inflammatory response of HGFs [12]. *Porphyromonas gingivalis* lipopolysaccharide (Pg-LPS) induced HGFs could secrete IL-1β, IL-6, IL-8, TNF-α and other inflammatory factors to activate the inflammatory response [13, 14]. IL-1β could promote the production of IL-6, and excessive IL-6 might lead to the destruction of connective tissue [15]. IL-6 was involved in the pathogenesis of periodontal disease by inducing osteoclastogenesis, tissue destruction and bone resorption [16], reducing the above inflammatory factors could reduce oxidative stress and inhibit the occurrence of inflammation [17]. Therefore, the inflammatory response of HGFs induced by Pg-LPS may play an important role in the occurrence and development of dental peri-implantitis.

Autophagy is a physiological process of programmed cell death, in which excessive protein and subcellular components were wrapped in autophagosomes and directed to lysosomes to digest and degrade [18]. The autophagosome formation is dependent on autophagy associated protein 5 (ATG5) and microtubule-associated protein light chain 3 (LC3) [19]. Beclin-1 was the first protein to be found to be associated with autophagy, which normally regulated autophagy in the form of the cytoplasmic solute [20]. Under normal physiological conditions, all cells undergone physiological autophagy to maintain the balance of physiological activities. Heat shock protein 90 (HSP90) was a highly conserved and very important chaperone protein in eukaryotes [21]. The most widely studied were HSP90β continuously expressed in cells, and HSP90α induced in response to cellular stress. HSP90α and HSP90β proteins were the result of gene replication, and 86% of the amino acid sequences were identical. HSP90α was encoded by the HSP90AA1 gene. In addition, research had shown that HSP90α was closely related to the occurrence and development of autophagy. Xiao et al. [22] found that high expression of HSP90AA1 gene could enhance the tolerance of osteosarcoma cells to chemotherapeutic drugs by inducing autophagy. Hu et al. [23] discovered that HSP90AA1 could promote the autophagy by directly interacting with AKT/mTOR signaling pathway. Autophagy could be mediated by NF-κB signaling pathways induce inflammation [24]. Our previous study found that HSP90AA1 might be a key gene in the development of peri-implantitis [25].

Therefore, we hypothesized that HSP90AA1 could promote the inflammatory response of HGFs induced by Pg-LPS by regulating autophagy. This study will further explore the role of HSP90AA1 gene in the development of peri-implantitis, and provide a potential target for clinical treatment of peri-implantitis.

## Materials and methods

### Cell culture and treatment

HGFs cells (HUM-iCell-m005, iCell Bioscience Inc, Shanghai, China) were cultured in DMEM medium containing 10% fetal bovine serum, at 5% CO_2_, 95% air and 37 °C constant temperature. HGFs were incubated with different concentrations of Pg-LPS (Invivogen, USA, purified from strain ATCC 33277) for 24 h to construct a model of HGFs in vitro. HGFs cells were divided into 4 groups: control group (normal HGFs without any treatment), Pg-LPS (0.1, 1, 10 μg/mL) treatment group (normal HGFs were co-cultured with Pg-LPS 0.1, 1, 10 μg/mL for 24 h).

### HSP90AA1-siRNA transfection

HGFs were stimulated with 1 μg/mL of Pg-LPS as the appropriate concentration. On the basis, siRNA targeting HSP90AA1 gene was designed, and HGFs were transfected together with transfection reagent to construct HGFs cell line with low expression of HSP90AA1. The HGFs cells were treated with Pg-LPS, Pg-LPS + siRNA empty vector, Pg-LPS + HSP90AA1-siRNA 1, 2 respectively. The optimal treatment of HSP90AA1-siRNA 1,2 was selected, and then experiments were performed with the addition of 3-MA (10 mmol/L) to verify that autophagy may mediate inflammatory responses through HSP90AA1 overexpression.

### CCK-8 assay

Logarithmic growth phase HGFs was treated with different concentrations of Pg-LPS (0.1, 1, 10 μg/mL) and cultured in 96 well plates for 24 h. Then 10 μL of CCK-8 solution was added to each well and incubated in the incubator. At last, absorbance was measured at 490 nm wavelength and cell viability was calculated. Six replicate cells were measured in parallel for each group.

### ELISA assay

200 μL cell suspension (5 × 10^5^/mL) was taken and added to 96 well plate for 24 h, and the supernatant was collected by centrifugation. The samples to be tested were obtained by filtration through a 0.22 μM microporous membrane. Enzyme labeled plates were first added to the tested samples, followed by enzyme-linked antibody (HRP) 100 μL incubated for 1 h. After washing the plate, the chromogenic solution was added for chromogenic reaction, followed by the addition of the termination solution. Finally, the OD value of each well was measured with an enzyme labeling instrument. The contents of the factors (IL-1β, IL-6, TNF-α) in the sample were calculated by comparing with the standard curve.

### Immunofluorescence assay

HGFs cells were planted on the plate and treated with drugs. The cells were fixed with 4% paraformaldehyde, and permeabilized with Triton X-100. PBS was rinsed and sealed with 5% BSA. Then anti-HSP90α (abcam, ab79849, 1:100), anti-HSP90β (abcam, ab53497, 1:100) antibody were added and incubated at 37 °C for 3 h. After washing with PBS, the secondary antibody IgG H & L (abcam, ab150080, 1:500) was incubated for 30 min. DAPI was added dropwise to dye the nucleus under dark conditions, and sealed slices, then the collected image was observed under the fluorescence microscope.

### ROS detection

HGFs cells (5 × 10^5^/ml) were cultured on a 6 well-plate. After drug treatment, the cells were washed with PBS solution and incubated in a medium containing 10 μM DCFH-DA for 45 min at 37 °C in dark environment. Then, DCFH-DA loaded cells were washed with PBS, and observed by fluorescence microscope. Fluorescence was measured at an excitation wavelength of 480 nm and an emission wavelength of 520 nm.

### Flow cytometry

The cells density were adjusted and the cells were incubated in a constant temperature incubator for 24 h. Cells were collected, centrifuged and resuspended with buffer. Then, 5 and 10 μL of PI and Annexin V reagents were added respectively, and incubated at room temperature for 10 min to detect the apoptosis rate. Three parallel assays were performed.

### Western blotting analysis

The cells were treated with lysate, cell samples were obtained by centrifugation, and the total protein concentration was determined by BCA method. 30 μg protein was separated by 15% SDS-PAGE electrophoresis and transferred to PVDF membrane. Then, it was sealed at room temperature with 5% skimmed milk powder for 2 h. The anti-HSP90AA1 (Affinity, BF0084, 1:1000), anti-LC3AB (Affinity, AF6139, 1:1000), anti-ATG5 (Affinity, DF6010, 1:1000), anti-Beclin1 (Affinity, AF0120, 1:2000), anti-NF-κB p65 (Affinity, AF5006, 1:1000), anti-Phospho-NF-kB p65 (Ser536) (Affinity, AF2006, 1:1000), β-actin (Affinity, AF7018, 1:5000) antibody were added and incubated at 4 °C overnight. Then, the secondary antibody was added and incubated at room temperature for 1.5 h. Finally, the chromogenic reaction was carried out, and the gray values of the bands were counted and the results were analyzed.

### RT-PCR analysis

Trizol reagent was used to extract total RNA from cells. CDNA was synthesized according to reverse transcription kit. Then the cDNA was used as the template and amplified by real-time fluorescence quantitative PCR. The expression of target genes was calculated by the 2^−△△Ct^ method, and GAPDH was used an internal reference.The primer sequences of gene were shown in Table 1.

**Table 1:** qPCR Primer sequences

Gene	Forward primer	Reverse primer
Human HSP90AA1	CACAGGTGAGACCAAGGACC	TTCCCCTAGTTTTCATGCCACA
Human β-actin	CATGTACGTTGCTATCCAGGC	CTCCTTAATGTCACGCACGAT

### Statistical analysis

By using SPSS 18.0 software, datas were analysied. One-way-ANOAY was used to analyze the multi-group difference, and then SNK test was used. Kruskal–Wallis H was used when the variance was uneven. The data were expressed mean ± SD, *P* < 0.05 suggested that the difference was statistically significant.

## Results

### Pg-LPS increased the levels of inflammatory cytokines and cellular ROS generation, but inhibited apoptosis

**Fig. 1:**
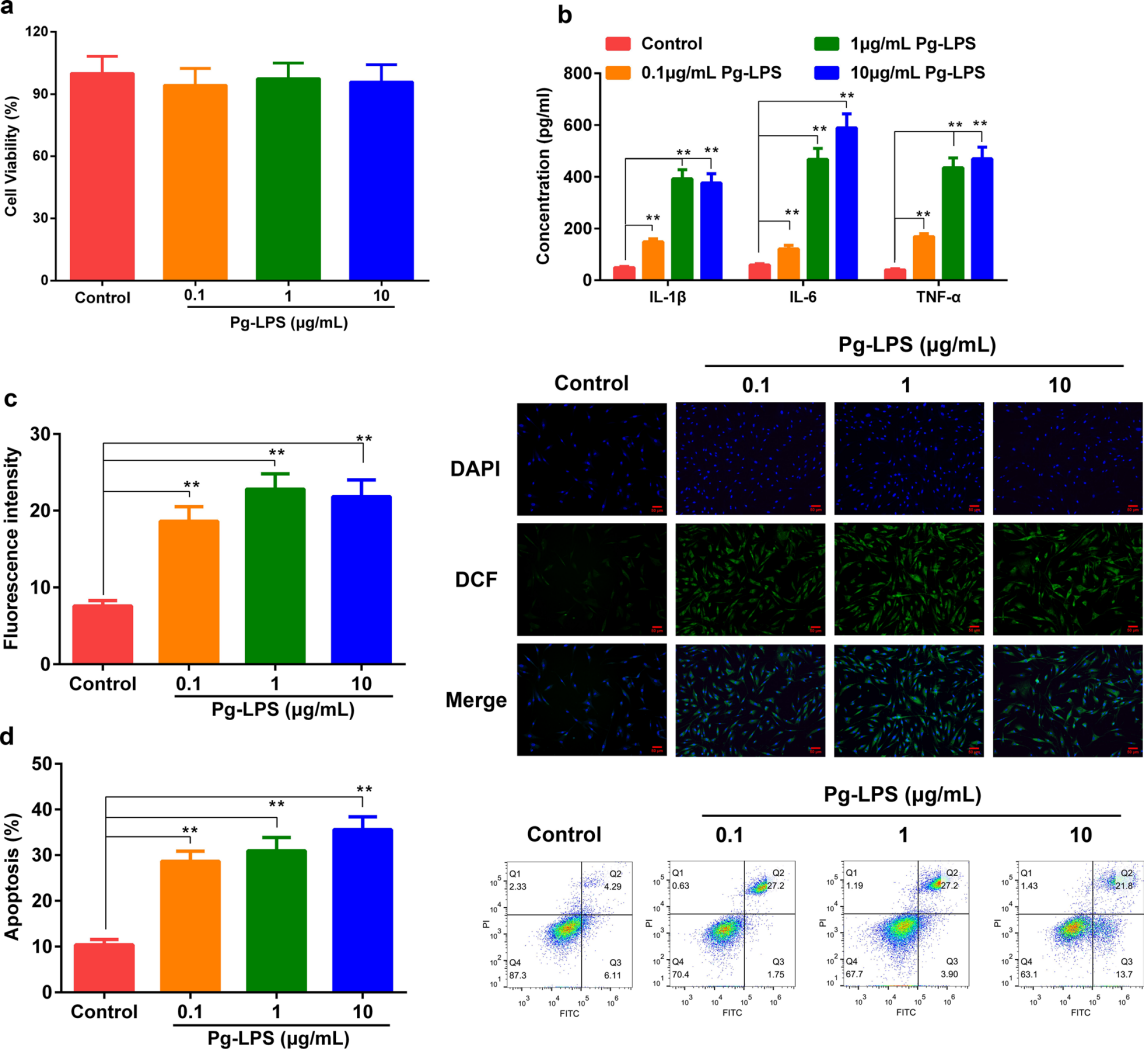
Pg-LPS did not affect the cell viability of HGFs, while increased the level of cytokines. HGFs was treated with different concentrations of PG-LPS (0.1, 1, 10 μg/mL) for 24 h, and detected the changes of cell viability (**a**) and inflammatory factors (**b**), cellular ROS generation (**c**) and apoptosis (**d**) were detected. Data were expressed as mean ± SD, n = 6. Compared to the control group, **P < 0.01. *Note*: human gingival fibroblasts, HGFs; *P. gingivalis* LPS, Pg-LPS

The effect of different concentrations of Pg-LPS (0.1, 1, 10 μg/mL) on the activity of HGFs were detected by CCK8 assay. The results showed that Pg-LPS had no effect on the viability of HGFs (Fig. 1a). In addition, the effects of different concentrations of Pg-LPS (0.1, 1, 10 μg/mL) on inflammatory factors in HGFs were detected by ELISA. As can be seen from Fig. 1b, compared with the control group, different concentrations of Pg-LPS significantly increased the levels of IL-1β, IL-6, and TNF-α. When the concentration of Pg-LPS was greater than 1 μg/mL, the levels of inflammatory factors were not significantly increased compared with 1 μg/m Pg-LPS. Immunofluorescence was used to detect the effect of different concentrations of Pg-LPS (0.1, 1, 10 μg/mL) on the ROS level of HGFs and the Flow cytometry assay was used to detect the apoptosis rate of HGFs. The results showed that different concentrations of Pg-LPS increased the level of ROS (Fig. 1c) and the apoptosis rate in HGFs (Fig. 1d).

### Pg-LPS treatment increased the expression of HSP90AA1 and autophagy related proteins

**Fig. 2:**
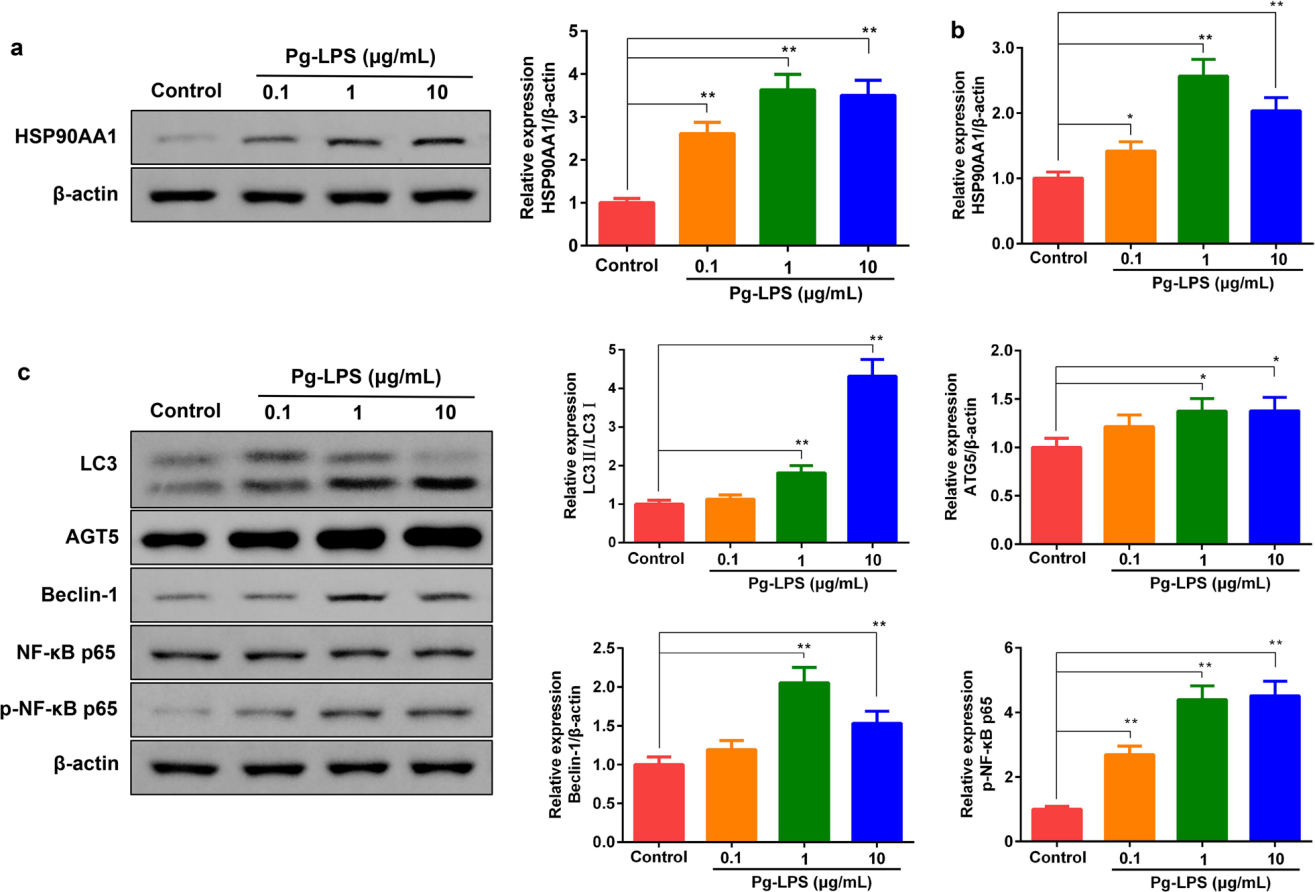
Pg-LPS treatment increased HSP90AA1 protein and gene level, and elevated the expressions of autophagy related protein in HGFs. **a, b** The changes of HSP90AA1 protein and gene level. **c** Pg-LPS (0.1, 1, 10 μg/mL) treatment increased the expression of autophagy related protein in HGFs. Data were expressed as mean ± SD, n = 3. Compared to the control group, *P < 0.05, **P < 0.01. *Note*: human gingival fibroblasts, HGFs; *P. gingivalis* LPS, Pg-LPS. The gels in **a**, **c** were cropped

As shown in Fig. 2a, Additional file 1-Fig 1, Fig.2b, compared with the control group, when the concentration of Pg-LPS was less than 10 μg/mL, the concentration of Pg-LPS dose dependently increased the levels of HSP90AA1 protein and HSP90AA1 mRNA. Compared with the 1 μg/mL Pg-LPS group, the effect of 10 μg/mL Pg-LPS was not significant. Morever, After Pg-LPS treatment, the expression of autophagy related proteins were measured by western blot. The results showed that compared with the control group, Pg-LPS treatment could significantly increase the protein levels of p-NF-κB p65/NF-κB p65, LC3 II/I, ATG5, and Beclin-1 (Fig. 2c, Additional file 1-Fig 2). Therefore, the in vitro inflammation model cell group (1 μg/mL Pg-LPS treatment group) with the highest expression of HSP90AA1 was selected for subsequent studies.

### HSP90AA1-siRNA reduced the expression of HSP90AA1 protein, mRNA, HSP90α, and HSP90β

**Fig. 3:**
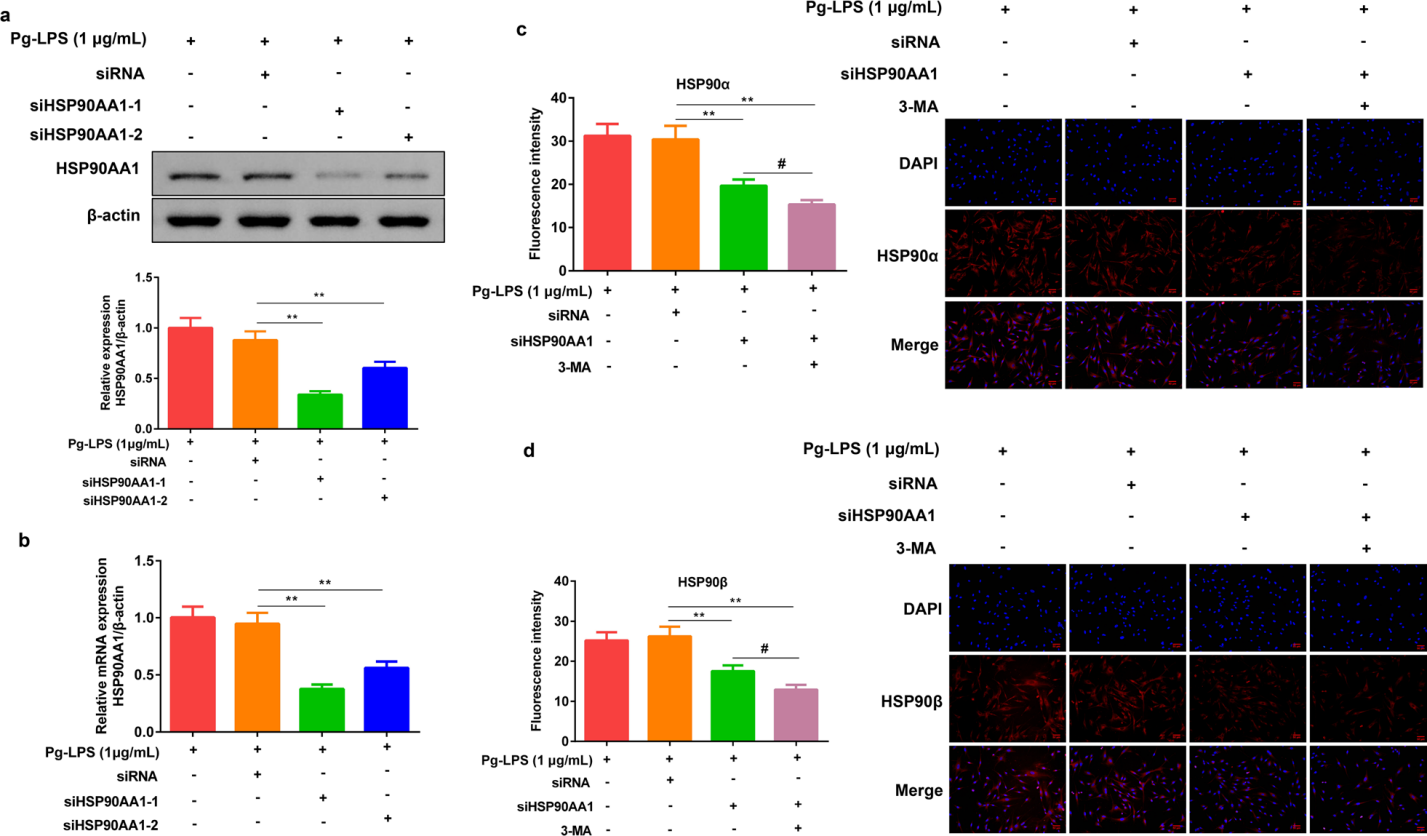
Construction of stable and low expression HGFs cell lines by HSP90AA1-siRNA transfection, and validation of the effectiveness of HSP90AA1-siRNA transfection. **a, b** The protein, mRNA level of HSP90AA1 in HGFs after transfection of HSP90AA1-siRNA. Immunofluorescence detected the protein content of HSP90α (**a**), HSP90β (**b**) after transfection of HSP90AA1-siRNA in HGF (×200). Data were expressed as mean ± SD, n = 3, 6. Compared to the Pg-LPS (1 μg/mL) + siRNA group, **P < 0.01; Compared to the Pg-LPS (1 μg/mL) + siHSP90AA1 group, ^#^*P* < 0.05. *Note*: human gingival fibroblasts, HGFs; *P. gingivalis* LPS (Pg-LPS, 1 μg/mL); HSP90AA1-siRNA, siHSP90AA1. The gels in **a** were cropped

HGFs with low expression of HSP90AA1 were constructed by transfecting HGFs with siRNA targeting HSP90AA1 gene together with transfection reagent. As shown in Fig. 3a, Additional file 1-Fig 3, Fig.3b, the results of qRT-PCT and Western Blot showed that the levels of HSP90AA1 protein and mRNA significantly decreased after transfection of HSP90AA1-siRNA. Therefore, the transfected HSP90AA1-siRNA group with the lower HSP90AA1 expression was selected for subsequent experiments. Futhermore, HSP90AA1 siRNA was transfected into HGFs cells, and then 3-MA was added to detect the expressions of HSP90α and HSP90β proteins in Pg-LPS induced HGFs, respectively. The results showed that the fluorescence intensity of HSP90α (Fig. 3c) and HSP90β (Fig. 3d) decreased significantly after transfection of HSP90AA1-siRNA. After adding 3-MA, the fluorescence intensity of HSP90α and HSP90β further decreased significantly.

### si HSP90AA1 reduced the levels of IL-1β, IL-6, and TNF-α

**Fig. 4:**
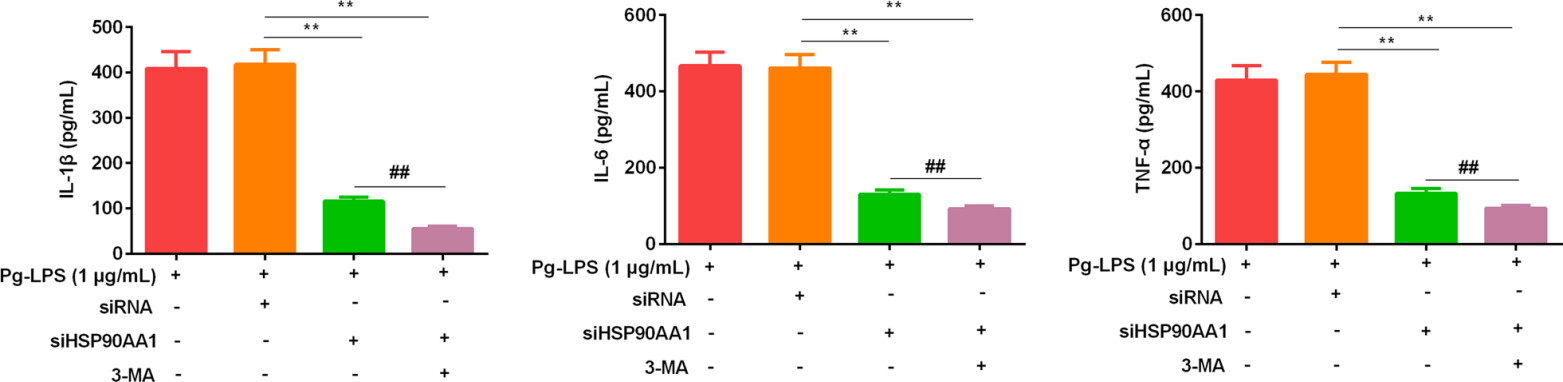
Effect of HSP90AA1-siRNA transfection on the contents of IL-1β, IL-6, and TNF-α. Data were expressed as mean ± SD, n = 3. Compared with the siRNA group, **P < 0.01; Compared to the Pg-LPS (1 μg/mL) + siHSP90AA1 group, ^##^*P* < 0.01. *Note*: human gingival fibroblasts, HGFs; *P. gingivalis* LPS (Pg-LPS, 1 μg/mL); HSP90AA1-siRNA, siHSP90AA1

ELISA assay detected the levels of inflammatory factors in the HSP90AA1-siRNA group and the HSP90AA1-siRNA combined with 3-MA group. The results (Fig. 4) found that the levels of IL-1β, IL-6, and TNF-α were significantly reduced after transfection of HSP90AA1-siRNA. The addition of 3-MA further reduced the levels of IL-1β, IL-6, and TNF-α, and these results indicated that HSP90AA1 could regulate the levels of inflammatory cytokines by mediating autophagy.

### HSP90AA1-siRNA transfection decreased cellular ROS generation, and inhibited the apoptosis

**Fig. 5:**
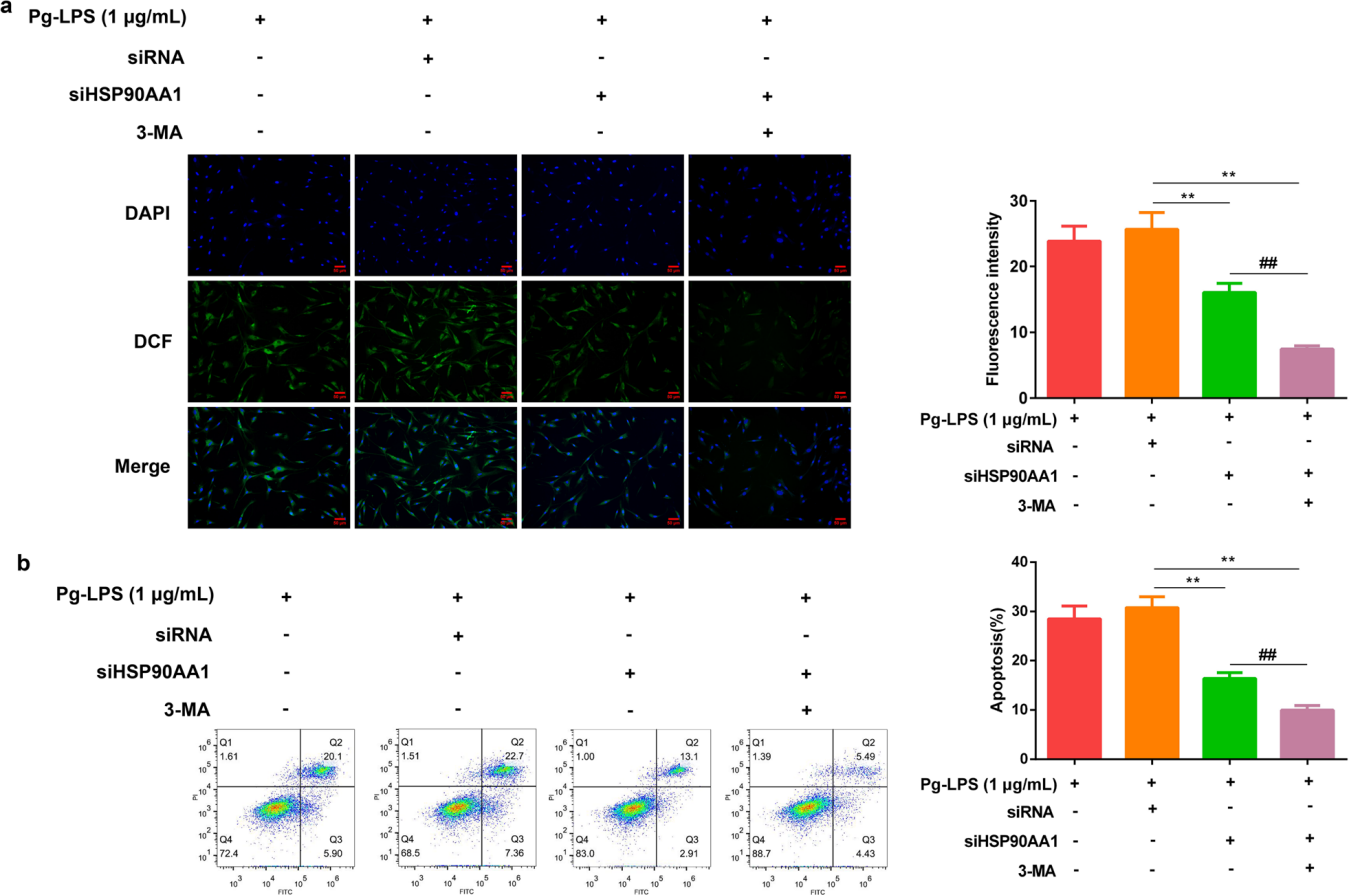
HSP90AA1-siRNA transfection decreased HGFs cellular ROS generation and inhibited apoptosis. **a** The Fluorescent ROS images (× 200), and fluorescence intensity analysis. **b** The apoptosis of HGFs was analyzed after transfection of HSP90AA1-siRNA and the addition of 3-MA. Data were expressed as mean ± SD, n = 3. Compared with the siRNA group, **P < 0.01; Compared to the Pg-LPS (1 μg/mL) + siHSP90AA1 group, ^##^*P* < 0.01. *Note*: human gingival fibroblasts, HGFs; *P. gingivalis* LPS (Pg-LPS, 1 μg/mL); HSP90AA1-siRNA, siHSP90AA1

The ROS levels of HGFs after transfection of HSP90AA1-siRNA and HSP90AA1-siRNA + 3-MA were detected by immunofluorescence assay, and it was found that the ROS level of HGFs was significantly decreased in the HSP90AA1-siRNA and HSP90AA1-siRNA + 3-MA group than those in the siRNA only transfected group (Fig. 5a). As shown in Fig. 5b, the number and rate of apoptotic cells in the transfected siHSP90AA1 group and HSP90AA1-siRNA + 3-MA were significantly lower than those in the siRNA only transfected group.

### siHSP90AA1 reduced the levels of autophagy and TLR related proteins

**Fig. 6:**
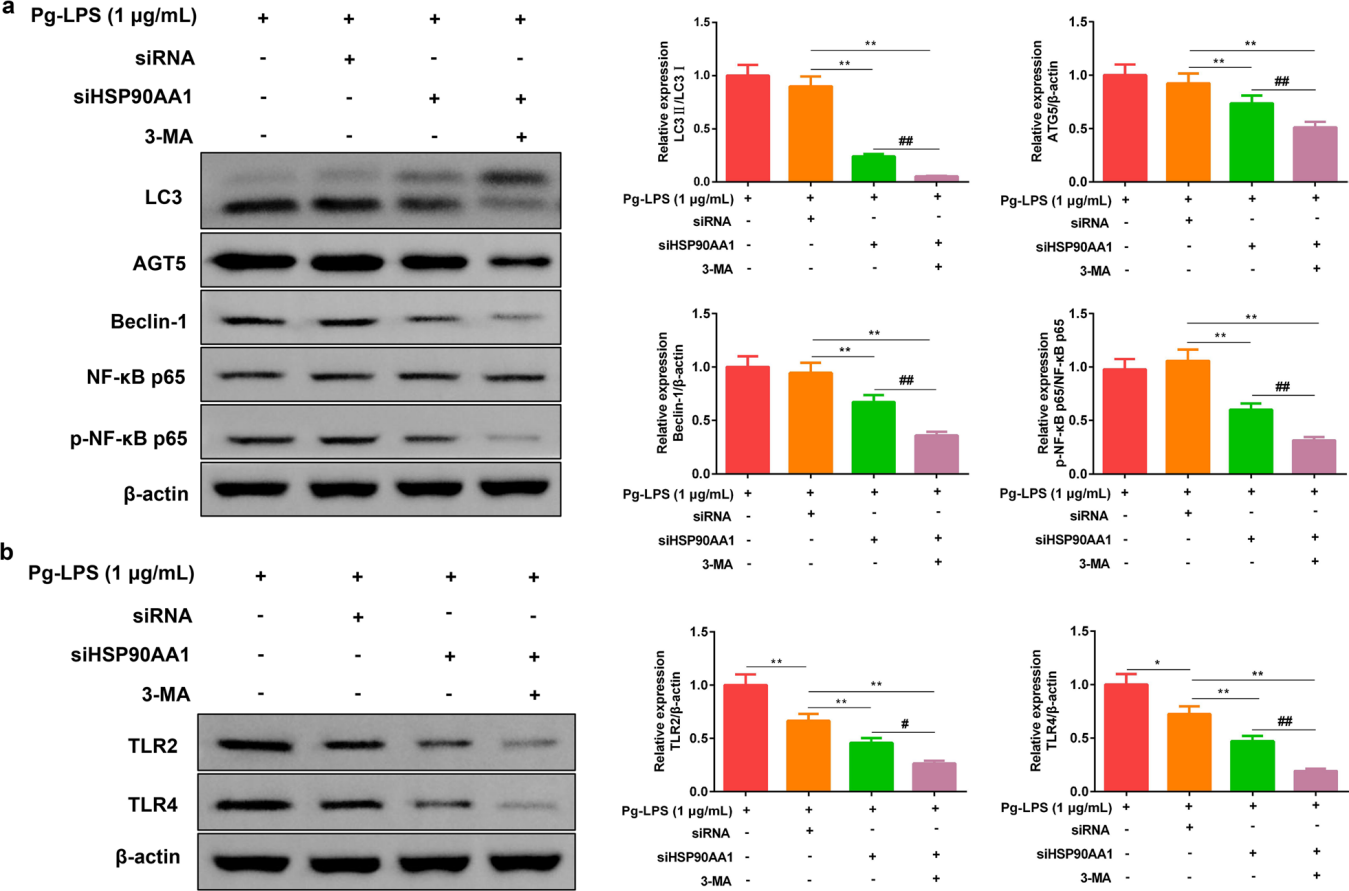
Low expression of HSP90AA1 reduced the levels of autophagy related protein and TLR related protein in HGFs. **a** The protein levels of LC3 II/I, Beclin-1, ATG5, and p-NF-κB p65/NF-κB p65. **b** The protein levels of TLR2 and TLR4. Data were expressed as mean ± SD, n = 3. Compared with the siRNA group, **P < 0.01; Compared to the Pg-LPS (1 μg/mL) + siHSP90AA1 group, ^##^*P* < 0.01. *Note*: human gingival fibroblasts, HGFs; *P. gingivalis* LPS (Pg-LPS, 1 μg/mL); HSP90AA1-siRNA, siHSP90AA1. The gels in **a**, **b** were cropped

After the cells were transfected with HSP90AA1-siRNA and HSP90AA1-siRNA + 3-MA, we detected the changes of autophagy related proteins and TLR-related proteins, and found that the protein levels of LC3 II/I, ATG5, Beclin-1, p-NF-κB p65/ NF-κB p65 and TLR2, TLR4 were evidently decreased (Fig. 6a, Additional file 1-Fig 4, Fig.6b, additional file 1-Fig 5). These fully verified that the HSP90AA1 gene could regulate the levels of autophagy related proteins and TLR-related proteins.

## Discussion

Peri-implantitis as a late complication usually occured after planting superstructure or dental prosthesis [26]. Although the survival rate of implant was very high (92.8–97.1%) [1], the complications such as peri-implant inflammation were easy to occur [27]. The occurrence of peri-implantitis has seriously affected the quality of life of dental implant patients. In this study, we investigated the effects of HSP90AA1 gene on autophagy, apoptosis and inflammatory of Pg-LPS induced HGFs. It was confirmed that HSP90AA1 could promote the Pg-LPS induced HGFs inflammatory response mainly by mediating cell autophagy. This study provided an effective target gene for the treatment of peri-implantitis.

HGFs could actively participate in immune and inflammatory responses [28]. The balance of cytokines regulated by immune response played an important role in the stabilization and development of inflammation [29]. Studies have shown that Pg-LPS could stimulate the expression of TNF-α, IL-1β, IL-6, and macrophage inflammatory protein (MIP)-1α in monocytes and macrophages [30, 31], and then induce osteoclast formation leading to alveolar bone loss [32]. Many studies have shown that ROS played an important role in inflammatory response. Bullon et al. [33] found that periodontal bacterial LPS stimulated HGFs to increase mitochondrial ROS. Liu et al*.* [34] considered that the secretion of IL-1β, IL-6, TNF-α in Pg-LPS induced HGFs is mediated by the interaction between P53 regulating ROS and ROS stimulating P53. Li et al*.* [35] found that ROS over produced after LPS treatment of HGF could induce HGFs to increase the levels of TNF- α, IL-1β, and IL-6 through MAPK and NF-κB pathway. In this study, we found that after treatment of HGFs with different concentrations of Pg-LPS, the proinflammatory factors levels of IL-6, IL-1β, TNF-α increased in a dose-dependent manner. We also confirmed that these inflammatory factors and intracellular ROS levels were significantly reduced after cells were transfected with HSP90AA1-siRNA, and the addition of autophagy inhibitor 3-MA further reduced the levels of inflammatory factors and intracellular ROS, suggesting that HSP90AA1-mediated autophagy of HGFs promotes inflammatory responses.

Accumulating evidences showed that autophagy was an important part of the innate and adaptive immunity of the host, and was associated with many inflammatory diseases [36]. Oxidative stress induced ROS has been shown to induce apoptosis and autophagy [37]. Bullon et al. [33] found that Pg-LPS lead to ROS mediated autophagy, suggesting that there was a link between autophagy and ROS in HGFs. which is consistent with Park's research, and the production of ROS promoted the transformation of LC3-I to LC3-II [38]. Both ATG5 and Beclin1 participated in the formation of autophagosome in autophagy signaling pathway [39]. It has been reported that the levels of RNA and protein expression of ATG5 increased in Pg-LPS induced HGFs [40]. El-Gowily have confirmed that up-regulation of Beclin-1 could produce disruptive autophagy [41]. Pg-LPS significantly increased the proteins levels of ATG5, Beclin1, and p-NF-κB p65/NF-κB p65 in HGFs, and the ratio of LC3-II/LC3-I was increased in dose-dependent manner. After transfection of HSP90AA1-siRNA, the response of these indicators were also reversed, accompanied by a decrease in the rate of apoptosis, which was further reversed by the addition of the autophagy inhibitor 3-MA. This indicated that HSP90AA1 was involved in regulating the levels of autophagy related proteins and cell apoptosis. Therefore, targeting the HSP90AA1 gene may be a potential target for regulating Pg-LPS induced HGFs inflammatory response.

Autophagy can sense and detect pathogens by recognizing pathogen related molecular patterns by pattern recognition receptor (PRRs) [42]. HSP90AA1 was the main pathogen receptor of bacterial LPS, and also a PRR component [43], it could bind with LPS, dengue virus [44], and avibirnavirus [45]. In recent years, some reports suggested that HSP90AA1 also existed on the cell surface [46]. Increased studies have used pathogen recognition to reveal the effect of pathogen infection on autophagy. It has been reported that influenza A virus (IAV) induced autophagy through the hemagglutinin (HA) combinated with HSP90AA1 on the cell surface [42]. Hu et al. [23] found that HSP90AA1 binded to avibirnavirus VP2 induced autophagy by inactivating the AKT-MTOR pathway. In this study, the addition of the autophagy inhibitor 3-MA better confirmed HSP90AA1 as a target for regulating autophagy in Pg-LPS stimulated HGFs.

In summary, this study indicated that HSP90AA1 could participate in regulating autophagy, and promoted Pg-LPS induced HGFs to produce inflammatory injury. This will provide a theoretical basis for us to explore the mechanism of HSP90AA1 involving in peri-implantitis. However, this study also has certain limitations. This study only established an inflammatory model of Pg-LPS induced HGFs in vitro to study the role of HSP90AA1 in regulating autophagy and promoting inflammation, while in vivo HSP90AA1 regulating autophagy and promoting peri-implantitis requires further exploration.

## Conclusion

In conclusion, in this study, we found that the levels of IL-1β, IL-6, TNF-α, p-NF-κB p65/NF-κB p65, LC3 II/I, ATG5, Beclin-1, and HSP90AA1 increased in Pg-LPS induced HGFs inflamation model, while these were reversed after transfection with HSP90AA1-siRNA. It suggested that HSP90AA1 promoted the inflammatory response of Pg-LPS induced HGFs by regulating autophagy, indicating that HSP90AA1 played an important role in peri-implantitis, and might provide a potential therapeutic target for clinical treatment.

## Supplementary Information

**Additional file 1:** Original image of the western blot protein bands.

## Data Availability

All data generated or analysed during this study are included in this published article and its supplementary information files.
