# Supplementary material for: HSP90AA1 promotes the inflammation in human gingival fibroblasts induced by Porphyromonas gingivalis lipopolysaccharide via regulating of autophagy
Source: BMC Oral Health. 2022 Aug 26;22:366. doi: 10.1186/s12903-022-02304-0 (PMC9419417; doi:10.1186/s12903-022-02304-0)
Supplement: Supplementary file 1 — Additional file 1: Original image of the western blot protein bands. [file 12903_2022_2304_MOESM1_ESM.pdf]

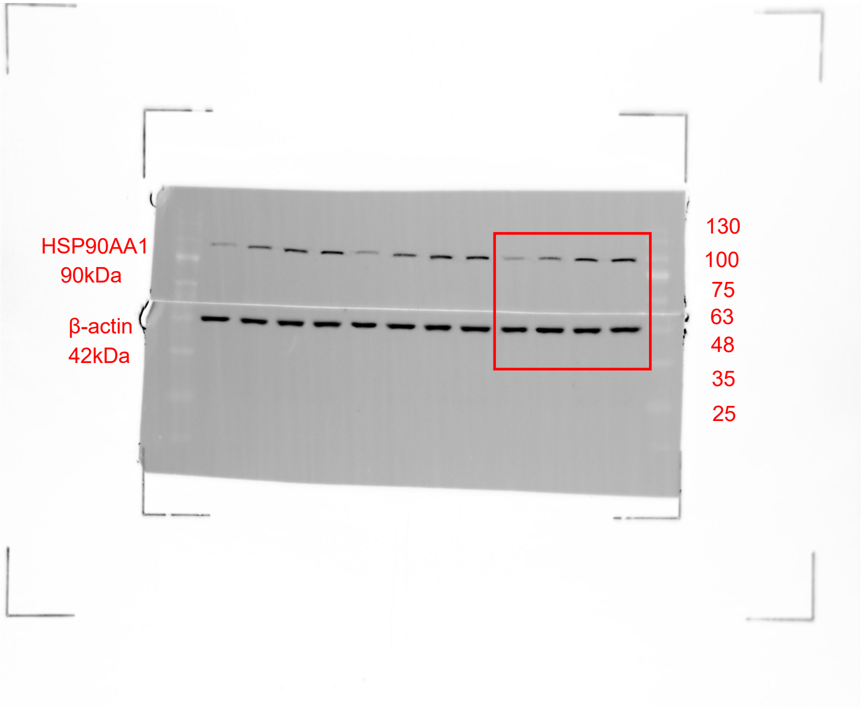

Manuscript related files Fig 1. The HSP90AA1 and β-actin protein bands are corresponding the original band of Fig. 2a in the manuscript. The target protein bands we selected should be in column 3 along with the internal control.

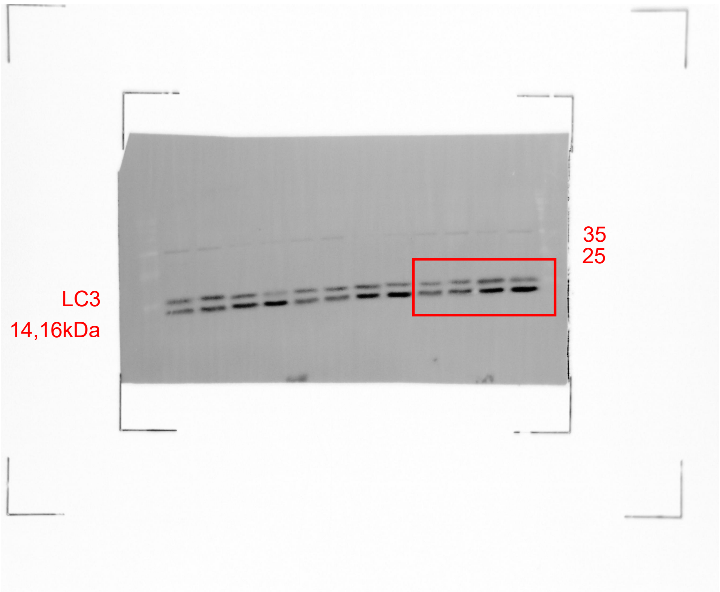

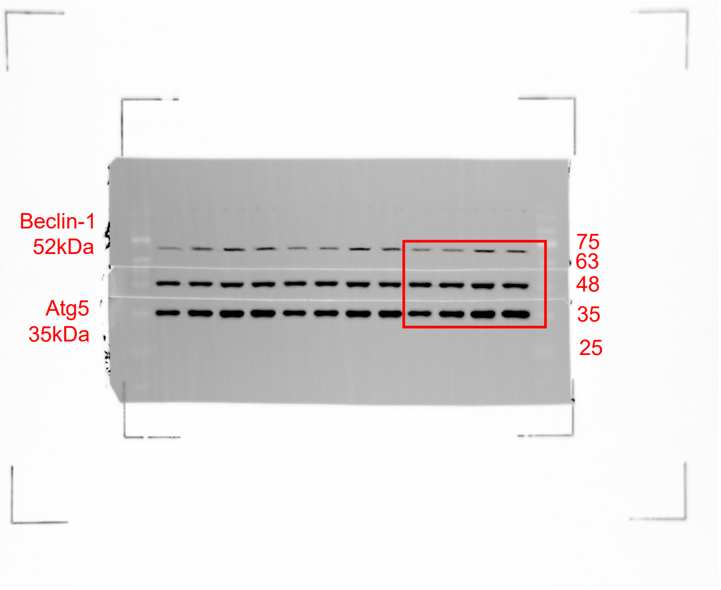

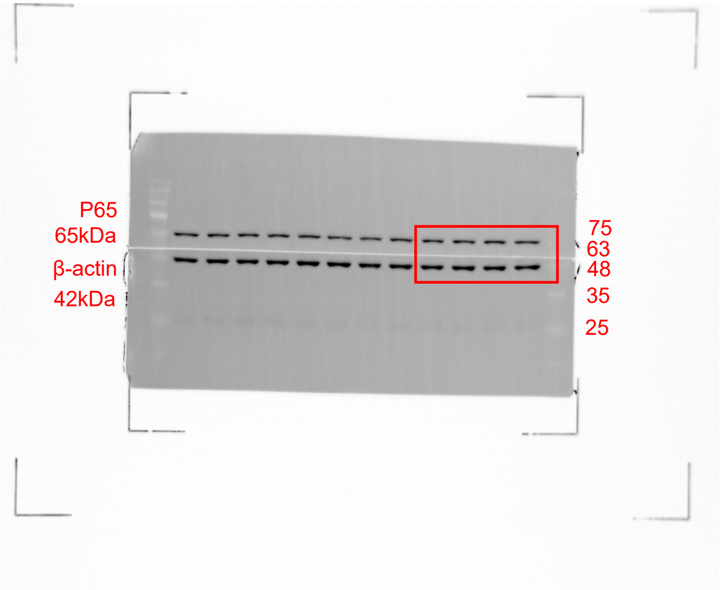

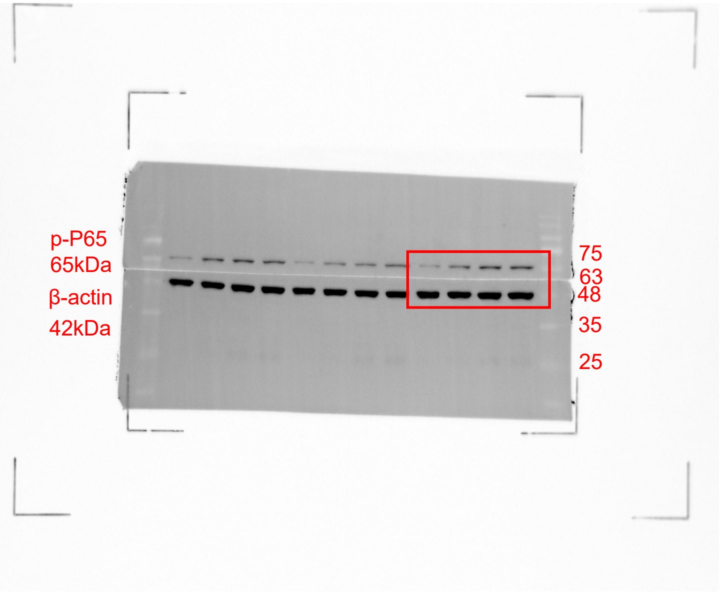

Manuscript related files Fig 2. The LC3, ATG5, Beclin-1, NF-κB, p-NF-κB, and β-actin protein bands are corresponding the original band of Fig. 2b in the manuscript. The target protein bands we selected should be in column 3 along with the internal control.

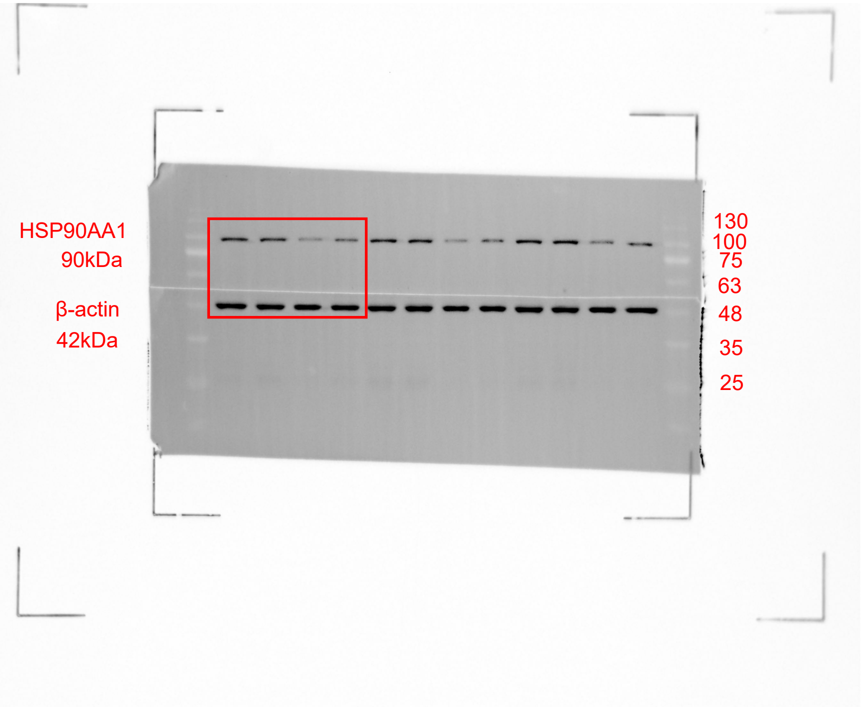

Manuscript related files Fig 3. The HSP90AA1 and β-actin protein bands are corresponding the original band of Fig. 3a in the manuscript. The target protein bands we selected should be in column 1 along with the internal control.

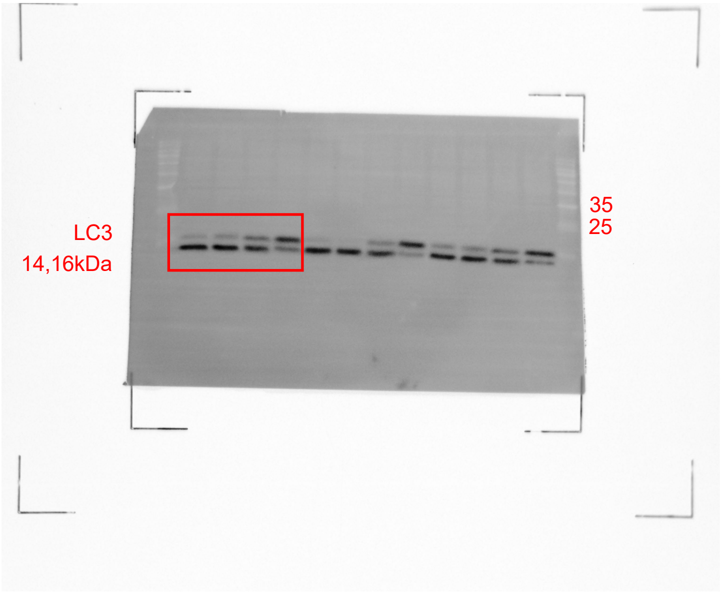

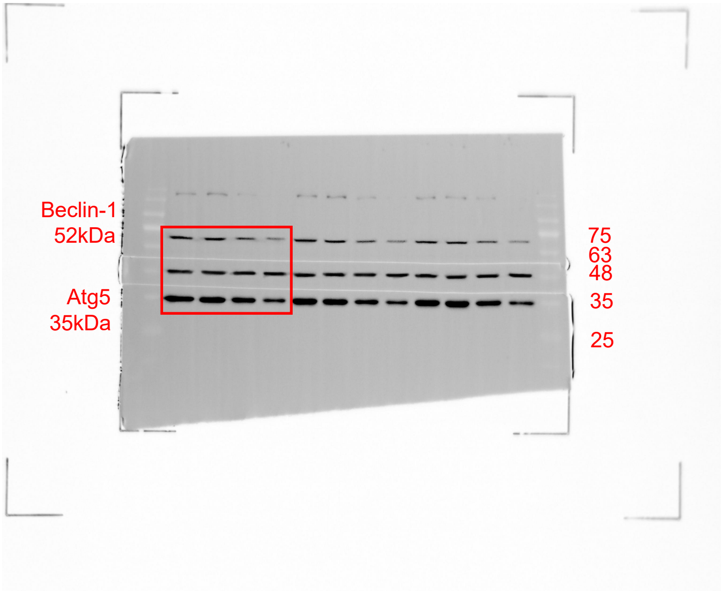

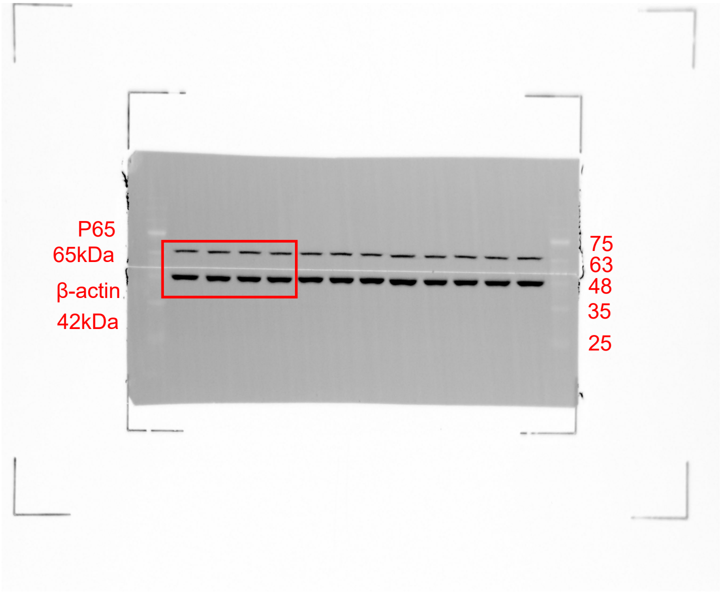

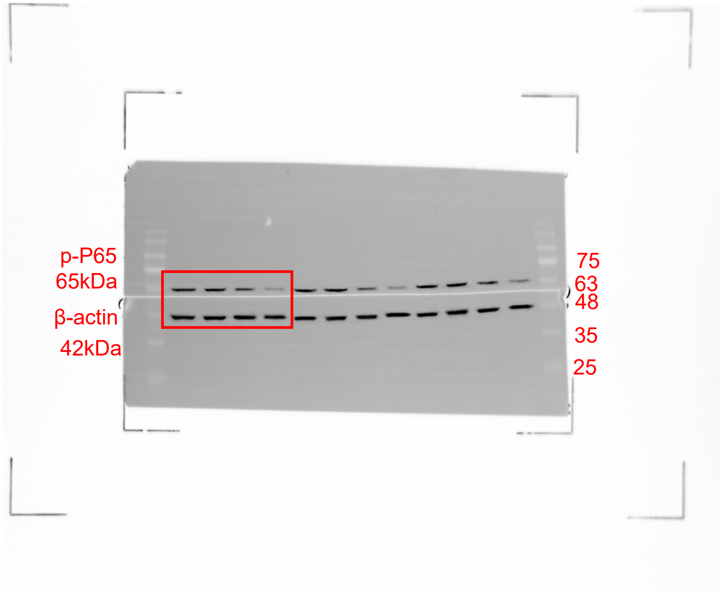

Manuscript related files Fig 4. The LC3, ATG5, Beclin-1, NF-κB, p-NF-κB, and β-actin protein bands are corresponding the original band of Fig. 6a in the manuscript. The target protein bands we selected should be in column 1 along with the internal control.

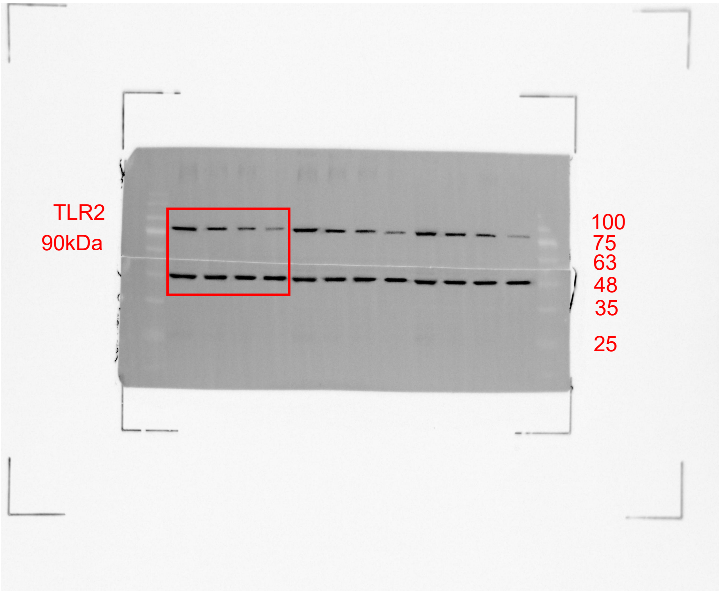

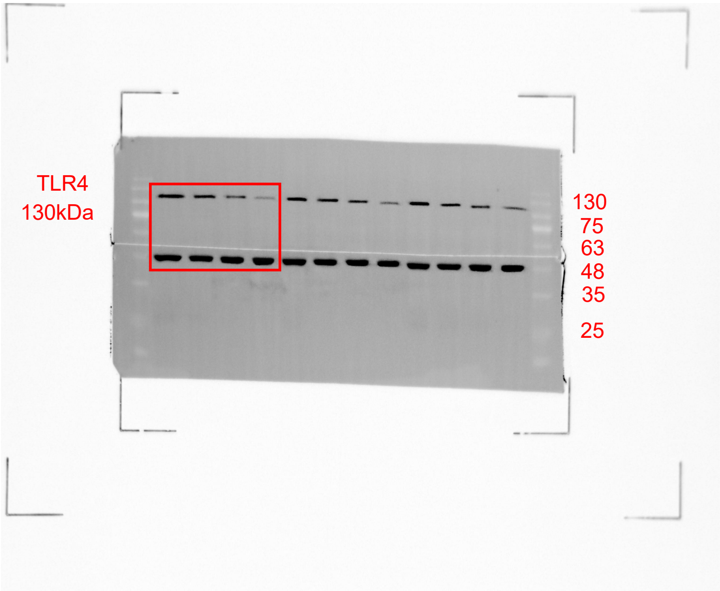

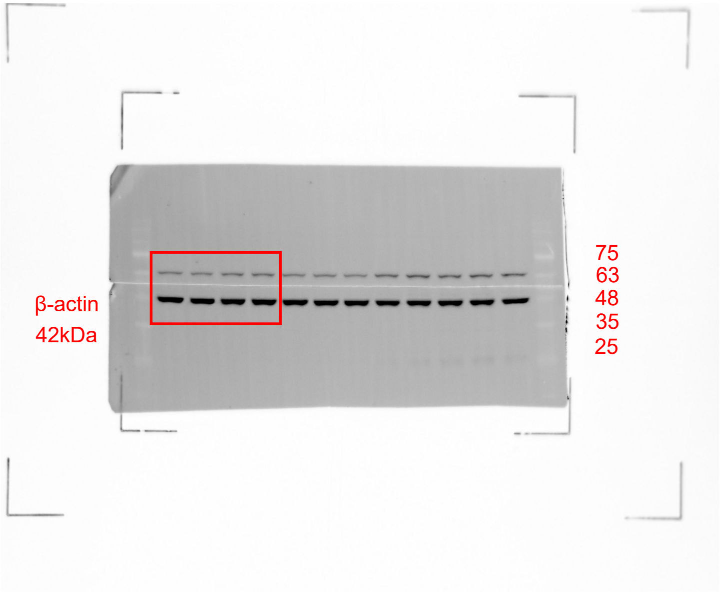

Manuscript related files Fig 5. The TLR2, TLR4, and β-actin protein bands are corresponding the original band of Fig. 6b in the manuscript. The target protein bands we selected should be in column 1 along with the internal control.
